# A holistic exploration of porous asphalt mixtures: From durability and permeability to low-temperature and functional properties

**DOI:** 10.1016/j.heliyon.2024.e41437

**Published:** 2024-12-29

**Authors:** Yue Zhou, Zhiqiang Cheng, Xiaoguang Zheng, Tao Wang, Xiaoliang Wu, Xiaoxiao Yu, Shengjia Xie

**Affiliations:** a Shanghai Road and Bridge Group Co. LTD, Shanghai, 200433, PR China; b Shanghai Municipal Engineering Design Institute (Group) Co.,LTD, PR China; c Anhui Provincial Key Laboratory of Urban Rail Transit Safety and Emergency Management, Hefei University, PR China; d Department of Highway and Railway Engineering, School of Civil Engineering, Beijing Jiaotong University, Beijing, 100044, PR China; e Shanghai Engineering Research Center of Green Pavement Materials, Shanghai, 200433, PR China

**Keywords:** Porous asphalt mixture, Raveling, Low-temperature performance, Permeability, Functional performance

## Abstract

This article provides a comprehensive review of the research progress on porous asphalt mixtures, focusing on their durability, permeability, low-temperature performance, and functionality. The study examines factors influencing the anti-raveling performance of porous asphalt, including modified asphalt binders, fibers, aggregates, traffic loads, and environmental conditions. It also discusses the impact of porosity, pore structure, and temperature variations on the low-temperature cracking resistance of porous asphalt. Furthermore, the article explores the permeability of porous asphalt mixtures, emphasizing the influence of pore characteristics, water flow directions, clogging processes, and pavement designs. The sound absorption and skid resistance properties of porous asphalt are also analyzed, highlighting the importance of porosity, texture depth, material properties, and water film thickness. The study concludes by proposing improvement measures and optimization suggestions for porous asphalt mixtures, providing valuable references for future research and applications. Finally, several future research topics for porous asphalt mixture are proposed. Further studies on porous asphalt pavement involves seeking performance balance, ensuring improvement without detriment. Researching factor interactions, building a model, exploring industrial waste use to cut costs, reduce pollution and promote its environmental application for diverse traffic and environment needs.

## 1 Introduction

Compared with dense-grade asphalt mixtures and stone mastic asphalt mixtures, porous asphalt mixture is a typical skeleton-pore structure. It is characterized by a higher amount of coarse aggregate than fine aggregate and filler and a porosity of greater than 15 %. The high porosity of the mixtures and the high internally connected pores allow rainfall to penetrate through the pavement promptly, reduce rainwater retention on the pavement surface significantly, and decrease the thickness of the water film accumulated on the porous pavement surface effectively. In addition, advantages of porous asphalt mixtures also include noise reduction, surface skid resistance, night visibility, low glare reflections, and reduction of splash and spray [1]. These advantages are beneficial for traffic safety, infrastructure economy, and sustainable environment. Thus, porous asphalt pavement, a form of drainage and quiet pavement, is widely applied to support the low-impact development (LID) philosophy, such as China's national sponge city program.

The high porosity of porous asphalt pavement, derived from the stone-on-stone contact structure, not only observably promotes permeability but also significantly weakens the mechanical properties and rutting resistance under the coupling efforts of traffic load and high temperature conditions. The surface areas of asphalt in porous asphalt mixtures are exposed more than those in traditional asphalt mixtures to environmental conditions, such as oxygen, moisture, and illumination, which accelerates the aging process of the asphalt. Due to asphalt aging, porous asphalt mixtures become brittle and prone to forming cracks on the pavement surface, resulting in water permeation and impaction of rheological, cohesive, and adhesive properties [[2], [3], [4]]. Clogging caused by dust, residual soil, and other surrounding vegetation leads to the direct loss of permeability and noise reduction of the pavement. Thus, the characteristic structure of porous asphalt pavement is disadvantageous for its service life.

Most studies on porous asphalt mixtures focus on the effects of additives and mixture gradation design on the mechanical properties, durability, and high-temperature rutting resistance of porous asphalt pavement. Common additives include high-performance asphalt modifiers (such as styrene-butadiene-styrene (SBS), rubber powder, and epoxy asphalt), reinforcing fibers (such as basalt fiber, cellulosic fiber, acrylic fiber, and polyester fiber) and rejuvenator, which are all widely used in porous asphalt mixtures [[5], [6], [7], [8], [9], [10], [11], [12]]. The crumb rubber enhances the high and low-temperature viscoelastic performance, storage stability and elastic recovery of porous asphalt, and also improves rutting resistance of porous asphalt mixture significantly. Moreover, the full-swelling asphalt has the highest complex modulus and zero-shear viscosity and the best rutting resistance. But compared with partial and full-degradation asphalt mixtures, it shows poorer adhesive and moisture damage resistance [8]. Epoxy modified porous asphalt mixture reinforced with Basalt fiber also s has good marshall stability and dynamic stability [9]. The rejuvenator is also verified to enhance the performance of the high viscosity modified porous asphalt. With the increase of rejuvenator, the rutting factor of recycled porous asphalt decreases and the creep compliance decreases first and then increases. The elastic recovery ability of aged porous asphalt is also improved with the addition of rejuvenator. Besides, the rejuvenatior remarkably improves the high temperature rutting resistance, water damage resistance [13,14]. The relationship between asphalt performance and the characteristics of mixture gradation design has also achieved fruitful results. Porous asphalt mixtures with similar volumetric parameters, including void ratio, coordination number, and packing density, can provide obviously different mechanical performance [[15], [16], [17]]. In addition, some porous materials studied to replace asphalt mixture are also studied. Such as the study on the feasibility of using stamp sand and ASA plastic composite to replace asphalt mixtures. The material has good high-temperature performance and strong resistance to rutting and water damage, meeting moisture susceptibility requirements. However, the low-temperature cracking resistance and anti-raveling performance are poorer than asphalt mixtures. Overall, the composite has certain advantages and is expected to be an environmentally friendly pavement material, yet improvements in low-temperature performance and aggregate bond are needed [18].

Research hotspots mainly concentrate on mix grading design, high-viscosity asphalt, mechanical properties, high-temperature deformation, durability, and fatigue properties, but functional, low-temperature, and raveling properties receive less attention. Therefore, summary articles about impact factors of raveling, low-temperature cracking resistance, permeability, sound absorption performance, and skid resistance have been reported. This paper also aims to summarize the influence factors of the above-mentioned performances and some performance improvement measures, hopefully providing a reference for future research directions and the development of porous asphalt pavement.

## 2 Raveling

Raveling is defined as the loss of individual aggregates due to abrasion caused by traffic loads, environmental conditions, or both. As pavement raveling can rapidly develop into pavement damage, which significantly threatens traffic safety and reduces the life expectancy of porous asphalt pavement, anti-raveling performance is generally identified as an important technical criterion of durability [19]. Compared with traditional asphalt mixtures, the anti-raveling performance of porous asphalt mixtures is more susceptible to the impact of traffic load and environmental factors due to their high porosity characteristic. The high porosity of porous pavement results in smaller contact areas among the aggregates, loss of adhesion between asphalt and aggregates, and fracture of the asphalt binder between aggregates. Pavement raveling is one of the serious distresses affecting porous asphalt mixtures [20].

### 2.1 Impact of environmental and traffic condition

Environmental factors affecting anti-raveling performance include heat, light, temperature, moisture, air, etc. As the open structure facilitates the passage of air and water through the interconnected voids, porous asphalt mixtures are prone to oxidation and aging, which leads to the erosion of the asphalt film and a reduction in the strength of the asphalt-aggregate bonding. The result of these aging defects eventually accelerates fragmentation, stripping, and raveling [21,22]. Moreover, porous asphalt material is susceptible to aging under ultraviolet radiation, leading to a rapid deterioration of raveling resistance. Therefore, the average service life of porous asphalt pavements is shortened to 10–12 years, far less than densely graded asphalt pavements with a service life of about 18 years [23].

Moisture is also considered an important factor in increasing the raveling of porous asphalt mixtures, whether in liquid state or vapor form [23]. The sensitivity of porous asphalt mixtures to raveling in wet conditions is obviously higher than that in dry conditions. Thus, the presence of moisture exacerbates the sensitivity to raveling of porous asphalt mixtures [24].

External temperature significantly increases the susceptibility of porous asphalt mixtures to raveling, especially at high temperatures. The raveling susceptibility results between 10 °C and 30 °C are similar, but there is a significant increase in raveling susceptibility at 50 °C. The impact of external temperature on the mechanical performance of the mixture is caused by changes occurring in the linear viscoelastic material properties of the mastics. When temperature changes, the resistance to failure of a viscoelastic material also changes [24].

Traffic factors such as traffic speed, load condition, and longitudinal forces raise the possibilities of raveling in porous asphalt mixtures [24]. Porous asphalt mixtures are more susceptible to raveling when the vehicles circulate at a lower speed 48 km/h than at a higher speed 113 km/h. The susceptibility to raveling is comparable between the mean load of 33.3 kN and the high load of 41.8 kN cases, and the susceptibility to raveling is reduced by 23–46 % at the low load of 24.9 kN. Porous asphalt mixtures are prone to raveling when the longitudinal force aligns with the kinematic friction force, specifically when the vehicle is in a braking state. Thus,in the case of low-to-medium speed pavements, areas where vehicles brake regularly, or zones with a high frequency of heavy vehicles, the susceptibility to raveling of porous asphalt pavement could be greater.

Although temperature and humidity among the environmental factors are difficult to control directly, it is feasible to utilize big data for analyzing traffic flow characteristics of specific road segments. This enables the anticipation of sections where raveling is likely to be exacerbated and the implementation of corresponding countermeasures. Moreover, innovative material designs can be employed to alleviate the adverse effects of external environmental factors on pavement raveling, such as modified asphalt binders, aggregates, fibers, other additives, and the design of mix proportion. The following discussion focuses on the impact of modified asphalt, fibers, and aggregates in order to improve the anti-raveling property of porous asphalt mixtures.

### 2.2 Impact of modified asphalt

Numerous studies have demonstrated that polymer-modified binders can enhance the adhesion between aggregates and asphalt binders, subsequently improving the performance of porous asphalt mixtures. Among the primary factors influencing the raveling resistance of porous asphalt mixtures are the content and type of binder.

In terms of content, the binder should be present in sufficient quantity to form a thick film on the aggregate particles, which helps to reduce oxidation and raveling. Regarding the type of binder, SBS copolymers and crumb rubber have been proven to enhance the durability of porous asphalt mixtures [25]. Zhang et al. [26] found that increasing the amount of high-viscosity SBS-modified binder reduces Cantabro loss and improves raveling resistance under immersion and freeze-thaw conditions.

Altan [27] investigated the effects of crumb rubber content and particle size on the raveling resistance of porous asphalt mixtures. The research revealed that the Cantabro loss rate decreases with a reduction in rubber content, and smaller rubber particle sizes tend to exhibit better abrasion resistance. The synthetic use of polymer-modified binders has been observed in porous asphalt mixtures.

Jiao et al. [28] and Sangiorgi et al. [29] evaluated the impact of crumb rubber on the performance of porous asphalt in comparison to SBS. The results indicated that crumb rubber not only improves Cantabro abrasion resistance but also enhances other performance aspects. One reason for this is that rubber-modified asphalt may increase the viscosity of porous asphalt and provide a higher asphalt content in the mixtures. Consequently, a substantial asphalt film surrounding the aggregates enhances the durability of the asphalt mixtures. However, it is inevitable that rubber-modified porous asphalt mixtures also have a negative impact on porosity and permeability [27,28,30].

In practical engineering applications, a comprehensive consideration of the balance and optimization of all aspects of performance is essential. It is not advisable to use certain modified asphalt in large quantities merely for the purpose of improving anti-raveling and durability. Instead, suitable addition proportions and modification techniques should be explored to ensure that the mixture attains favorable levels in multiple crucial performance indices.

### 2.3 Impact of fibers

Fibers are the most commonly used stabilizing additive in porous asphalt mixtures, serving to retain the asphalt, prevent asphalt leakage, increase film thickness, and enhance anti-raveling performance. Porous asphalt mixtures without fibers exhibit poorer anti-raveling resistance [31,32]. Common fibers added to porous asphalt mixtures include organic fibers (such as lignin fibers, polymer fibers, and polyester fibers) and inorganic fibers (such as basalt fibers, carbon fibers, asbestos fibers, and diatomite fibers).

While fibers significantly enhance the abrasion resistance of porous asphalt mixtures, their efficiency varies among different types. Gupta found that 9 mm chopped aramid fibers enhanced the abrasion resistance of porous asphalt mixtures under dry conditions but reduced it under wet conditions [33]. When aramid pulp and glass hybrid fibers were used together as additives in porous asphalt mixtures [34], the resistance of the mixtures was improved under both dry and wet conditions. Zhang [12] used three sizes of cured carbon fiber composite materials (CCFCMs) from aerospace production lines to enhance the strength and durability of porous asphalt mixtures. It was found that the fine-sized CCFCMs had no impact on Cantabro abrasion resistance but increased other performances such as indirect tensile (IDT) strength and rutting resistance. Altan [35] investigated the benefits of adding basalt fiber and found that the addition of basalt fiber significantly reduced the loss of Cantabro particles, but excessive basalt fibers weakened the cohesion in the bitumen matrix and the adhesion between bitumen and aggregate. Zhang et al. [31] evaluated the effect of four fillers, including lignin fiber, basalt fiber, polyester fiber, and polyacrylonitrile fiber, on the performance of porous asphalt mixtures. It was concluded that lignin fiber and basalt fiber showed the worst anti-raveling property, followed by polyacrylonitrile fiber and polyester fiber. Regardless of aging condition, polyester fibers enhanced the raveling resistance of porous asphalt mixtures, but lignin fibers had an adverse effect on abrasion performance [36]. The poor tensile strength of lignin fiber-modified asphalt mixtures limited its application.

**Fig. 1:**
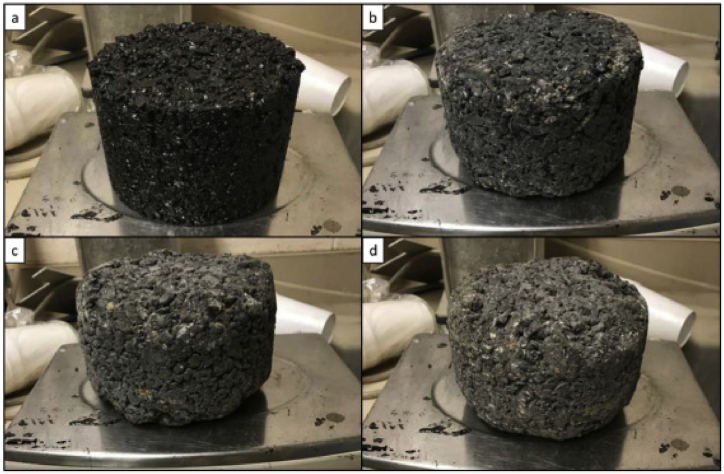
Cantabro abrasion of control PHMA: (a) Initial, (b) after 100 Revolutions, (c) 200 Revolutions, and (d) 300 Revolutions [37].

**Fig. 2:**
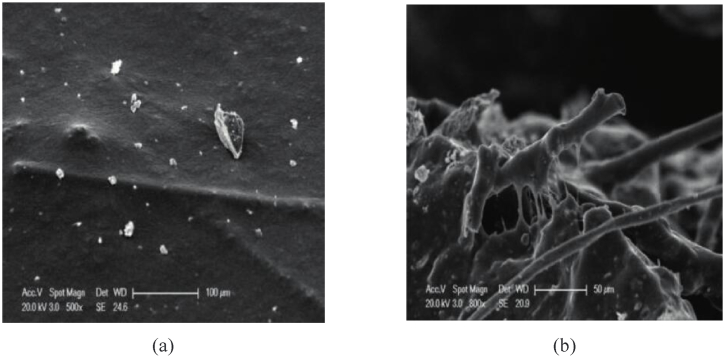
SEM micrographs of bonding interface between the fiber and asphalt. (a) Asphalt wrapped with chopped basalt fiber; (b) distribution of fibers in asphalt [10].

Moreover, excessive fiber content caused agglomeration and reduced the Cantabro loss of porous asphalt mixtures. There are two main reasons to explain the negative impact of excessive fibers. Firstly, most fibers have a filamentous appearance and tend to form agglomerates during blending with asphalt. Secondly, excessive fibers absorb most of the asphalt, resulting in insufficient adhesion among aggregates. Nevertheless, the mechanism of action of fibers in porous asphalt mixtures is still not clear. As shown in Fig. 1, Fig. 2, it is observed through SEM micrographs that the randomly distributed fibers form a three-dimensional spatial network structure by entangling with each other and absorbing some free asphalt [10]. This three-dimensional network structure compensates for the inner defects of the asphalt mixtures and improves the integrity of the structure, which prevents crack propagation [10]. In addition, high SBS content in modified binder in porous asphalt may exhibit a “masking” phenomenon in unaged conditions and covers the enhancement effect of fibers, **but long-term aging makes the “masking” effect of high-content SBS Modified porous asphalt mixtures disappear and the fiber enhancement more obvious** [36].

### 2.4 Impact of aggregates

Different aggregates exhibit varying physical and chemical properties, thus the selection of aggregates influences the raveling performance of porous asphalt mixtures [38]. The acid-base properties of aggregates mainly depend on their silica content, such as acid aggregates containing more than 65 % silica, and alkaline aggregates containing less than 53 % silica. The bonding between alkaline aggregates, such as limestone and diabase, and acidic asphalt is stronger than the bonding between acidic aggregates, such as granite and basalt, and acidic asphalt. On the other hand, anti-stripping agents such as hydrated lime, cement, and diatomite are recommended to improve the adhesion between acid aggregates and asphalt [39].

**Fig. 3:**
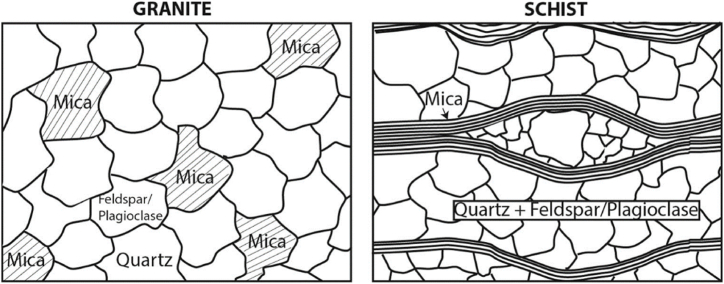
Scheme of granite and schist internal structure [40].

Besides acid-base properties, other physical properties including water absorption coefficient and Los Angeles Abrasion also impact the raveling of porous asphalt mixtures. As for the water absorption coefficient of aggregates, a coefficient lower than 2 % predicts good quality and excellent resistance to raveling. Concerning Los Angeles Abrasion, different types of aggregates share similar mineralogy but still have different internal structures (as shown in Fig. 3) and different mechanical strengths [40]. Thus, aggregates with good mechanical strength play a key role in determining the raveling property of porous asphalt mixtures. It is verified that aggregates with low Los Angeles Abrasion values in porous asphalt mixtures produce good anti-raveling properties, regardless of the type of modified asphalt used [41].

Aggregate gradation also impacts the structural integrity and raveling resistance of porous asphalt mixtures. The coarse aggregate content should be high as it increases the porosity of the mixture. And the fine aggregate content should be low to increase the contact area among aggregates, prevent the separation of coarse aggregates, and avoid forming closed air voids. In addition, increasing the content of fine aggregate passing through a 4.75 mm sieve generally results in a decrease in raveling [42].

As a promising and environmentally friendly technology, steel slags are used to improve the mechanical and functional properties of porous asphalt mixtures [43]. Steel slags are easily obtained as a byproduct from steel industries and have been verified as a substitute for natural aggregates in porous asphalt pavement. Compared with natural aggregates, steel slag aggregates have better physical properties such as lower abrasion loss, lower flakiness, lower elongation index, and higher angularity coefficient [44]. Many research studies have been conducted on incorporating steel slags into conventional porous asphalt pavement for the purpose of improving its performance. The steel slags used as aggregates in porous asphalt mixtures can meet the Cantabro abrasion loss requirements in optimal dosage and decrease the susceptibility to raveling [[44], [45], [46]].

In summary, the major factors affecting the raveling resistance of porous asphalt pavement are modified asphalt binders, fibers, aggregates, and additives. SBS modified asphalt, rubber modified asphalt, or both are generally employed to enhance the interaction between the binder and aggregates, inhibiting the segregation of materials. Fibers are usually incorporated with modified binders in porous asphalt mixtures to improve the integrity of the structure and extend the service life of porous asphalt pavements. The reasonable type and dosage of fibers are chosen based on the requirements. The aggregate, especially recycled and environmentally friendly materials including steel slag, is also extremely important for the durability and frictional resistance of porous asphalt mixtures.

## 3 Low-temperature performance

Porous asphalt mixtures have a porosity of at least 18 %, which is much higher than that of traditional asphalt mixtures. As a result, they are prone to low-temperature cracks, especially in cold regions, which restricts their application. At normal temperatures, asphalt mixtures exhibit viscoelastic plastic behavior and gradually transition from an elastic state to a plastic state as the temperature decreases. Low-temperature cracks occur when porous asphalt mixtures fail to release internal thermal stress in a timely manner under external low-temperature conditions. Therefore, both internal and external factors affect the low-temperature performance of porous asphalt mixtures.

### 3.1 Impact of environmental temperature

**Fig. 4:**
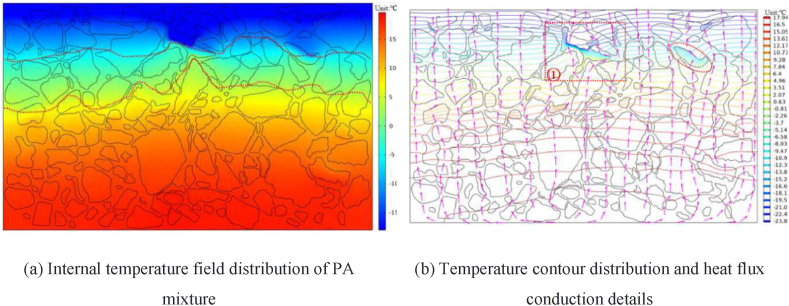
Temperature distribution of porous asphalt mixture (pores filled with air) [48].

**Fig. 5:**
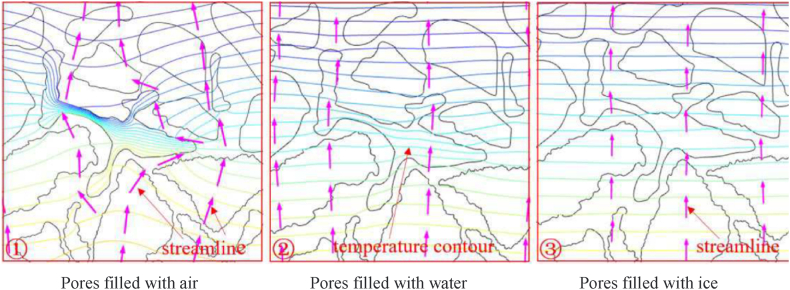
Details of temperature distribution at pores [48].

The rapid variation of environmental temperature results in the formation of a temperature gradient between the internal structure of asphalt mixtures and the surrounding environment [47]. As the depth of porous asphalt mixtures increases, the influence of environmental temperature decreases, revealing the hysteretic nature of the mixture's internal temperature. Additionally, the significantly different thermal conductivity of water, ice, and air leads to varying internal temperatures in porous asphalt mixtures when their pores are filled with these mediums [48]. Fig. 4, Fig. 5 effectively illustrate the relationship between the thermal transfer mediums and the internal temperature of the mixtures. It is evident that the isotherm distribution within the porous asphalt mixture filled with air is more uneven compared to that filled with water or ice. The presence of water or ice in the pores can easily degrade the low-temperature performance and potentially shorten the service life of porous asphalt mixtures. Therefore, both environmental temperature and the presence of pore water have a crucial impact on the internal structure of porous asphalt mixtures. Moreover, establishing a comprehensive temperature prediction model considering multiple environmental factors would be beneficial for timely preventive maintenance.

### 3.2 Impact of dynamic water erosion and frost heave

**Fig. 6:**
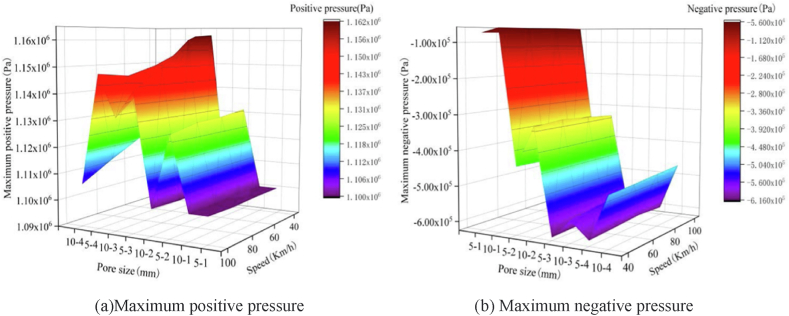
Trend of maximum positive and negative pressure changes [47].

The negative impacts of water on the low-temperature performance of porous asphalt mixtures primarily encompass dynamic water erosion and frost heave damage. During winter, when snow melts incompletely, a portion of the free water may infiltrate into the interior of the porous pavement. This, coupled with the simultaneous action of vehicle loads and low speeds on the pavement, can lead to "scouring" and "pumping" effects due to changes in instantaneous pore pressure. As illustrated in Fig. 6, under conditions of a vehicle speed of 40 km/h and a load of 157 kN, the maximum and minimum pore water pressures are recorded as 1.162 MPa and −0.616 MPa, respectively. These pressures have the potential to enlarge and loosen the pores within the porous asphalt pavement. The persistent scouring and pumping action of water can ultimately weaken the adhesion between asphalt and aggregate, resulting in the formation of additional connecting pores.

Given that porous asphalt mixtures possess a greater number of pores and connected pores compared to traditional asphalt mixtures, water can more easily penetrate the internal structure. As the temperature continues to decrease, ice crystal frost heave damage becomes a concern. The development of ice crystals is influenced by ambient temperature, duration, and pore shape. Lower ambient temperatures, longer durations, and smaller pore sizes all contribute to the growth of ice crystals. Typically, ice crystal frost heave damage first appears on the road surface due to the absence of confining pressure. The volume expansion caused by ice crystal frost heave exacerbates the extrusion deformation between aggregates and asphalt, providing a pathway for moisture to infiltrate the interior of the pavement structure and ultimately causing structural damage. Consequently, it is advisable to select asphalt with low temperature sensitivity and good deformation resistance for construction in low-temperature environments.

### 3.3 Impact of pore size

**Fig. 7:**
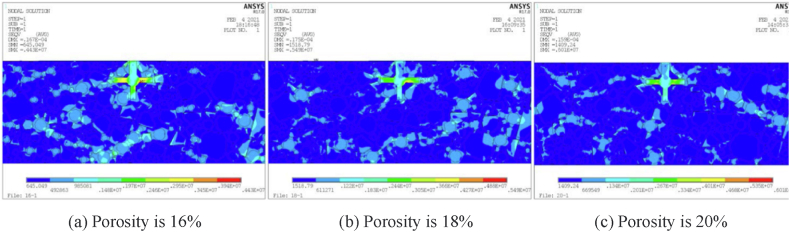
Structural stress cloud diagram [47].

**Fig. 8:**
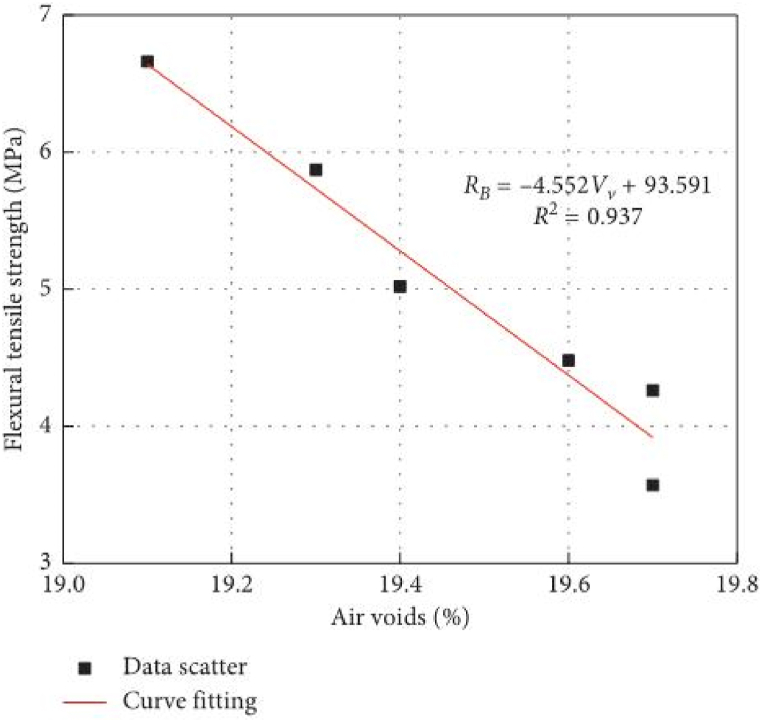
Relation between flexural tensile strength and air voids [49].

The degree of frost heave damage caused by ice crystals in porous asphalt mixtures is determined by both the number of pores and external temperatures. Based on MATLAB simulations and finite element analysis, it is evident that the frost heaving force of ice crystals increases with porosity and decreases with ambient temperature, as depicted in Fig. 7 [47]. Additionally, Wu [49] conducted a three-point bending test and confirmed that the flexural tensile strength of porous asphalt mixtures diminishes as porosity increases, as shown in Fig. 8. Both modeling analysis and experimental research consistently demonstrate that porous asphalt pavement with greater porosity exhibits reduced resistance to low-temperature cracking. Furthermore, in cold regions, porous asphalt pavements with larger porosity are more prone to developing issues such as looseness and potholes. Consequently, it is advisable to utilize porous asphalt mixtures with smaller porosity in areas that experience frozen conditions.

### 3.4 Impact of adding fibers and steel slags

Recently, numerous studies have contributed to enhancing the low-temperature properties of porous asphalt mixtures. Common methods to address pavement issues, including low-temperature cracking and reducing temperature sensitivity, involve using fibers and steel slags.

**Fig. 9:**
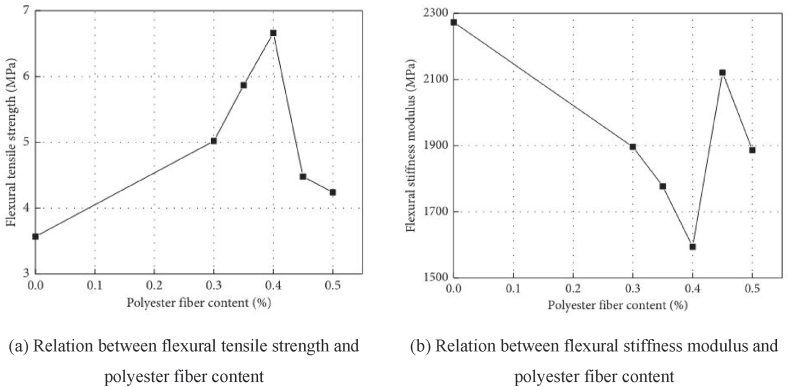
Tree-point bending test [49].

**Fig. 10:**
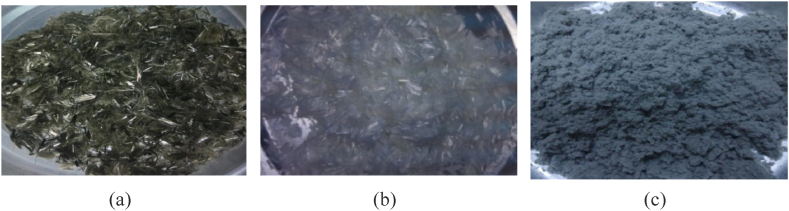
Basic appearance of three kinds of fibers: (a) Basalt fiber; (b) Polyester fiber; (c) Lignin fiber.

Generally, the addition of fibers not only improves the viscosity of asphalt but also enhances the cracking resistance of porous asphalt mixtures, ultimately extending their low-temperature lifespan. The fibers distribute evenly, adsorb excess free asphalt, and form a three-dimensional network structure within the porous asphalt mixtures. This structure increases the thickness of the asphalt film and improves the low-temperature cracking resistance of the porous asphalt. Wu found that the flexural tensile strength of porous asphalt mixtures initially increases and then decreases with an increase in polyester fiber content, while the flexural stiffness modulus exhibits the opposite trend [49]. The low-temperature shrinkage stress strength and anti-cracking energy of porous asphalt mixtures reach their peak values when the optimum polyester fiber content is 0.4 %. Qi studied the effect of basalt fiber content on the damage of porous asphalt mixtures after multiple freeze-thaw cycles [50]. The three-point low-temperature bending results in Fig. 9 showed that mixtures with 2 % basalt fiber content have a higher trabecula stiffness modulus than mixtures without basalt fiber, indicating that basalt fibers can improve the low-temperature ductility of porous asphalt mixtures. Low-temperature bending creep data revealed that porous asphalt mixtures with 2 % basalt fiber exhibit the minimum strain increase among three types of mixtures, indicating that basalt fiber is beneficial for the low-temperature deformation resistance of porous asphalt mixtures. Furthermore, Wang's research verified that chopped basalt fibers of 9 mm and 12 mm generally improve the low-temperature performance of porous asphalt. As shown in Fig. 10 [10,51], the reinforcement mechanism involves the fibers combining with asphalt and distributing in a three-dimensional network structure within the porous asphalt mixture, as observed through scanning electron microscopy (SEM). In addition, Zhang used cured carbon fiber composite materials in porous asphalt mixtures and reached similar conclusions [52]. The above studies all indicate that fibers have advantages for the low-temperature performance of porous asphalt mixtures. However, fibers include organic fibers such as lignin fibers and polyester fibers, as well as inorganic fibers such as basalt fibers, which exhibit different properties. Guo [51] studied the effects of lignin fiber, polyester fiber, and basalt fiber on porous asphalt mixture performance and concluded that basalt fiber with a length of 6 mm and a content of 0.4 % contributed to the improvement of engineering properties, including low-temperature crack resistance.

**Fig. 11:**
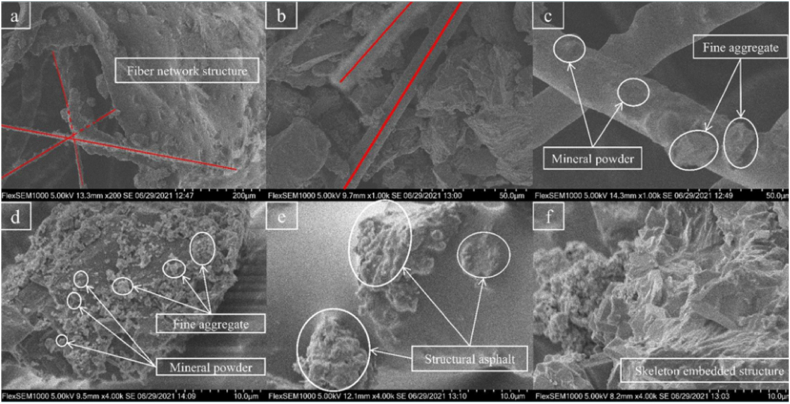
SEM microstructure is shown in (a)∼(f) [54].

Some researchers have studied the impact of steel slag on the low-temperature performance of porous asphalt mixture. Liu [48] indicated that the quality of aggregate played a vital role in the low-temperature crack resistance of porous asphalt mixture, as poor-quality aggregates were prone to stress concentration at low temperatures and could eventually lead to frost heave failure. Compared with natural coarse aggregates, steel slags have been proved to have superior mechanical properties and good adhesion with asphalt. Zhu [43] found that the cracking behavior of porous asphalt mixture at low temperatures could be significantly improved by adding 100 % steel slag, compared with other steel slag contents, by employing acoustic emission technology. However, Lou [53] found that the bending strain decreased with the increase of steel slag content, demonstrating that the porous structure of steel slag would absorb more free asphalt, resulting in a decrease in adhesion between aggregates. This conclusion seems to contradict Zhu's research [43]. To further improve the low-temperature crack resistance of porous asphalt mixture, Zhang [54] prepared porous asphalt mixture with different dosages of steel slags and polyester fibers and found that a 50 % steel slag replacement rate and 0.45 % polyester fiber content in porous asphalt mixture resulted in the best low-temperature performance. Chai [55] studied the low-temperature crack resistance of porous asphalt mixture with styrene-butadiene-styrene (SBS) polymer modified bitumen as the binder, steel slag as the aggregate, and crumb rubber and basalt fiber as modifiers. He found that 100 % steel slag replacement in modified porous asphalt mixture displayed the best low-temperature performance under the combined effect of rubber and basalt fiber. As shown in Fig. 11, the addition of steel slag promotes the transformation of more free asphalt into structural asphalt, which reduces the moisture sensitivity of the asphalt mixture and improves its durability. It also increases the asphalt film thickness on the aggregate surface and strengthens the gradation. The incorporation of polyester fiber reduces the temperature sensitivity of asphalt, improves the performance of asphalt mortar, and enhances the fracture toughness and crack resistance of the mixture.

Generally, appropriate amounts of steel slag, fiber, and asphalt can collectively enhance the low temperature ultimate tensile stress at different temperatures. The adhesion of aggregates can be enhanced by the chemical reaction between the alkalinity of steel slag and the acidity of asphalt. The interlock structure of steel slag and the three-dimensional network structure of fiber can reduce the brittleness of porous asphalt mixture, make the internal force distribution more uniform, and ultimately enhance the low temperature crack resistance. Besides, the quality of steel slag dramatically affects the low temperature performance of porous asphalt mixture.

### 3.5 Impact of clod recycling asphalt

The research on the low-temperature performance of cold recycled asphalt mixtures (CIR) is of crucial significance for ensuring the service performance of roads in cold environments. Through relevant experimental studies, it has been found that the low-temperature performance of CIR mixtures is affected by multiple factors. The Disk-Shaped Compact Tension (DCT) tests indicate that freeze-thaw cycles can reduce the fracture energy, thereby affecting the low-temperature cracking resistance. Meanwhile, the use of asphalt emulsion in the asphalt binder can improve the low-temperature flexibility but also sacrifice the high-temperature stability to some extent. In addition, the role of pavement structure design and environmental factors cannot be overlooked. A reasonable structure can relieve low-temperature stress, while cold and humid environments exacerbate the risk of low-temperature damage [56,57].

Based on this, future research can focus on optimizing the formula and dosage of asphalt emulsion, seeking a balance between low-temperature performance and other performances. At the same time, further exploration of the influence of additives on low-temperature performance and the development of more accurate performance prediction models are needed to better guide the application of CIR mixtures in low-temperature environments.

In summary, porous asphalt mixtures are prone to low-temperature cracks especially in cold regions, restricting the application. The low-temperature performance of porous asphalt mixtures is affected by multiple factors. Environmental temperature changes lead to temperature gradients, and the medium in pores affects internal temperature distribution, with water or ice presence impairing low-temperature performance. Dynamic water erosion and frost heave damage cannot be ignored. In winter, water infiltration under vehicle loads damages the pore structure, and ice crystal frost heave exacerbates structural damage at low temperatures. Pore size affects cracking resistance, and high porosity is prone to low-temperature cracks and other problems. Adding fibers and steel slags can improve low-temperature performance. Fibers enhance viscosity and cracking resistance, with different fibers having different effects, and the role of steel slag is controversial but appropriate use can enhance performance. The low-temperature performance of cold recycled asphalt mixtures is affected by freeze-thaw cycles, asphalt emulsion, etc. Future research needs to optimize the formula, study the influence of additives, and establish prediction models to guide application.

## 4 Permeability

Porous asphalt pavement can not only effectively remove rainwater from the pavement surface but also reduce the burden on the drainage system [58]. However, due to the impact of external traffic conditions and the environment, the large pores of porous asphalt pavement are easily blocked in its early stages of operation, resulting in a gradual attenuation of its permeability function.

### 4.1 Impact of air voids

**Fig. 12:**
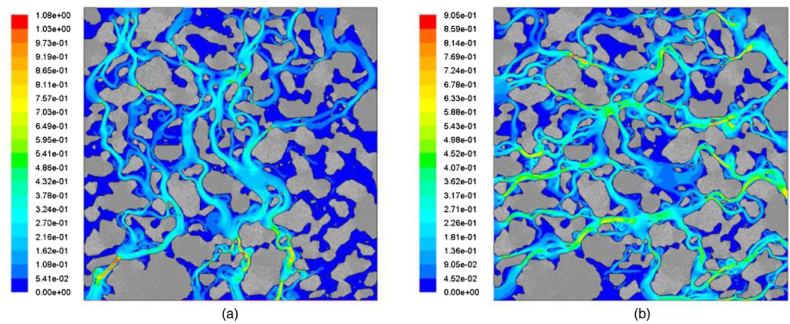
Fluid speed contour (m/s) under (a) vertical; and (b) horizontal flow [61].

Some studies on the permeability characteristics of porous asphalt mixtures have primarily focused on air voids. Zhang's research [59] indicated that increasing the particle size of aggregates would raise the average single void area and the percentage of total big voids, contributing to the formation of more interconnected voids and improving the permeability of porous asphalt mixtures. The results revealed that the void area and void length jointly affect the drainage performance. Water seepage prioritizes areas where the void area is large and the void length is sufficient. If the interconnected void is narrow and resembles a throat, water seepage will become saturated and difficult to flow out, as shown in Fig. 12. Voids with a large area and short length would form closed voids, which do not benefit either drainage performance or mechanical performance. Even if different porous asphalt mixtures have the same porosity, their permeability will not be exactly the same. Mixtures with a smaller nominal maximum particle size exhibit void characteristics with more void numbers, less total pore area, fewer big voids, and shorter void lengths, resulting in fewer interconnected voids and worse permeability [60,61]. Furthermore, the 9.5 mm sieve passing percentage of aggregate is considered a key factor in drainage performance. As the equivalent pore diameter affects the drainage capacity of porous asphalt pavement, and the 9.5 mm sieve passing percentage has the greatest influence on the equivalent pore diameter, increasing the 9.5 mm sieve passing percentage would optimize the microstructure parameters and, in turn, improve the drainage capacity of porous asphalt pavement.

### 4.2 Impact of water flow directions

**Fig. 13:**
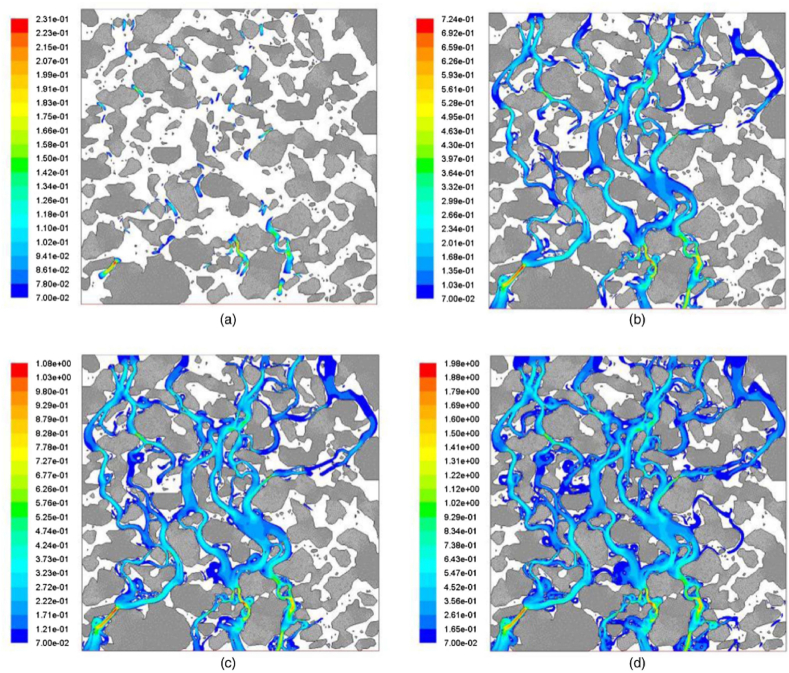
Fluid speed contour in the vertical direction at different inlet pressure: (a) 100 Pa; (b) 500 Pa; (c) 1000 Pa; and (d) 3000 Pa [61].

The difference in drainage performance between horizontal and vertical directions is also significant in the practical application of porous asphalt mixtures. Research has found that the equivalent void size in the horizontal direction is small and even, and the number of voids in the horizontal direction is numerous. By contrast, the characteristic of vertical voids is coarser and fewer [61]. Furthermore, the void bending (curvature) and the hydraulic diameter of the void have a marked impact on the seepage of porous asphalt mixtures. In general, the water flow rate decreases as the void curvature increases, and a void with a larger hydraulic diameter means that the shear resistance from the wall of the void is decreased and water flow is easier to discharge. It is also found that the void curvature in the horizontal direction is greater than that in the vertical direction, and increasing the porosity is helpful to reduce the void bending degree. Meanwhile, the hydraulic diameter in the horizontal direction is lower than that in the vertical direction, and the growth of porosity contributes to seepage. Besides, it is noted that the water pressure distributes unevenly in the vertical and horizontal directions, and the flow pathways increase with the increase of water pressure, as shown in Fig. 13. As revealed by the results, the horizontal flow is greater than the vertical flow under the same porosity, and the decrease of porosity would reduce the flow velocity. Meng [62] explored the variation of permeability in the clogging process of porous asphalt mixtures and drew a similar conclusion that the transverse seepage velocity is greater than the longitudinal seepage velocity, whether the porous asphalt mixture is clogged by particles or not. The permeability of porous asphalt mixtures varies from non-Darcy to Darcy permeability, and the seepage velocity after clogging is obviously lower than that before clogging. However, Ji [63] reported that the vertical and transverse permeability seemed to have no difference and exhibited good consistency.

### 4.3 Impact of clogging process

**Fig. 14:**
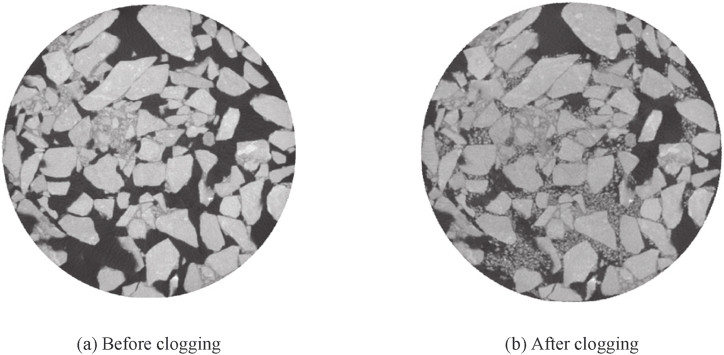
Comparison of tomographic images before and after clogging [62].

The water seepage performance of porous asphalt pavement is more sensitive to particle clogging compared with other types of asphalt pavements. The clogging of porous asphalt pavement consists of sediment particles of different sizes that exist in rainwater runoff, as shown in Fig. 14. Regarding the clogging process, Meng [62] used computerized tomography to capture tomographic images of porous asphalt mixtures and found that the clogging process experiences four stages in time sequence: fast clogging, clogging recovery, gradual clogging, and clogging acceleration. The research also indicated that transverse clogging lacks a clogging recovery stage compared with vertical clogging. Additionally, Hu [55] reconstructed a virtual porous asphalt pavement using a three-dimensional DEM-CFD model and deduced the following four stages in the pavement under rainfall: rapid clogging, slow clogging, partial recovery, and clogging stability.

**Fig. 15:**
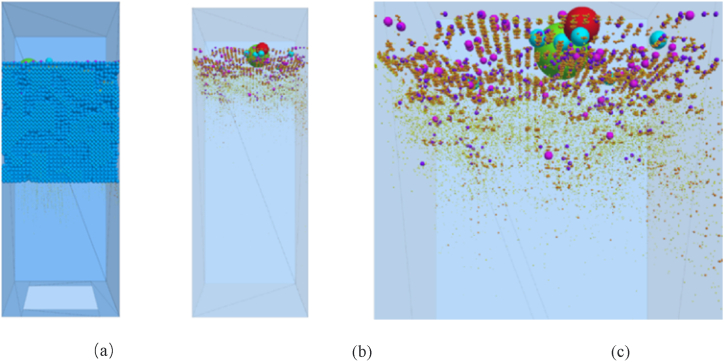
Distribution of clogging materials in the porous asphalt pavement under gravity: (a) simulation results of clogging test; (b) the accumulation of clogging material in transparent specimen; (c) closer inspection of clogging materials in the specimen [55].

Characterizing the clogging process of porous asphalt mixtures is also affected by water head height, gradation composition, and service times. As revealed by research [62], the impact of water on clogging particles increases with the rise of water head height. Furthermore, the deposition depth of particles is closely related to the gradation type of the asphalt mixture. The deposition position of clogging particles is deeper, and the clogging acceleration stage appears faster, with the increase of coarse aggregate content [55,64]. In the case of double-layer porous asphalt pavement composed of a top layer with small voids and a bottom layer with large voids, it is observed that the weight of deposited clogging particles in the top layer is 8.3 times greater than that in the bottom layer of the pavement, as shown in Fig. 15. Additionally, an increase in water flow results in a slight decrease in the mass of clogging particles in the bottom layer of the double-layer pavement, as some particles pass through the bottom layer. It is also found that the clogging degree of double-layer porous asphalt pavement is lower than that of single-layer porous asphalt pavement. Furthermore, factors such as traffic loading and service times both influence the permeability of porous asphalt pavement. Taking double-layer porous asphalt pavement as an example, accumulated traffic loading causes the rapid reduction of interconnected porosity in the early service life. The interconnected porosity decreases to below 15 %, and the water seepage performance is also severely degraded after 20 years of service [55].

### 4.4 Impact of pavement designs

Apart from the material characteristics of porous asphalt mixtures, the geometric design of the pavement, including cross slopes, thickness, and pavement width, also impacts drainage performance. The water discharge capacity of porous asphalt pavement increases with the rise of the cross slope gradient, although the growth trend gradually slows. The critical drainage strength of porous asphalt pavement is almost a quadratic function of drainage length as the drainage layer thickness increases. Research also verifies that an increase in porous asphalt pavement thickness is beneficial for improving permeability capacity, whether for double-layer or single-layer porous asphalt pavement [63,55]. Furthermore, drainage length plays a crucial role in the drainage performance of porous asphalt mixtures. The critical drainage strength of the pavement increases significantly, especially in the early stages of decreasing drainage length.

In summary, permeability, as a vital function of porous asphalt pavement, is easily affected by various factors, leading to clogging and the loss of water drainage performance. The permeability function of porous asphalt mixtures increases with porosity. And improving the 9.5 mm sieve passing percentage plays an important role in increasing interconnected voids and drainage performance. Besides, appropriately increasing the amount of coarse aggregates and reducing the amount of fine aggregates will enhance the drainage performance of porous asphalt mixtures. Additionally, from a road design perspective, it is feasible to improve cross slopes and pavement layer thickness when aiming to enhance water drainage performance. Furthermore, clogging particles and external heavy traffic conditions deeply affect the seepage performance of porous asphalt pavement, resulting in rapid degeneration of seepage performance within a short service life. While the permeability of porous asphalt pavement can be improved through the aforementioned techniques, other properties such as mechanical properties and durability may be compromised. Therefore, it is essential to ensure that other properties are maintained while improving the permeability characteristic of porous asphalt pavement.

## 5 Sound absorption performance

### 5.1 Impact of air voids

**Fig. 16:**
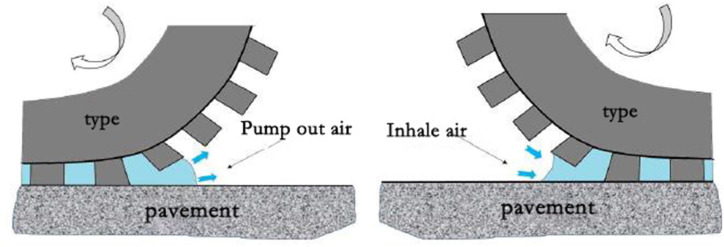
Air pumping effect [**61**].

Pavement noise is caused by friction between vehicle tires and the pavement surface, specifically, air is repeatedly pumped out and inhaled between the tire and pavement. Compared with other types of asphalt concrete and cement concrete, the large pore structure of porous asphalt mixtures contributes to absorbing and dispersing sound energy. Thus, porous asphalt pavement has become a potential means of sound absorption due to its low cost and source noise reduction capabilities [65]. However, the noise reduction effect of porous asphalt mixtures will gradually decrease over time, mainly due to pore blockage, pavement looseness, and aged asphalt, as shown in Fig. 16 [66]. In order to prolong the noise reduction service life of porous asphalt mixtures, research on various impact factors on their sound absorption performance is necessary.

**Fig. 17:**
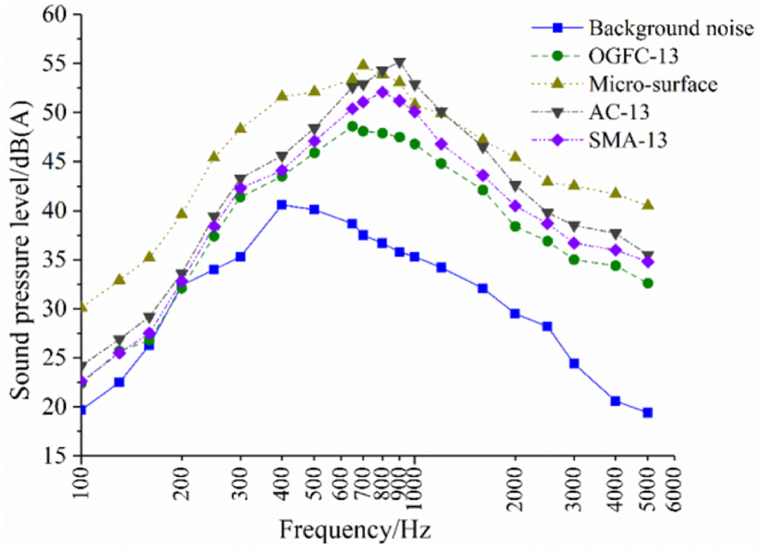
Noise frequency diagram of different pavement type [66].

Pore characteristics are the main factors affecting the sound absorption performance of porous asphalt mixtures. When sound waves act on porous asphalt pavement, some of the sound waves are reflected, while the rest continue along the internally connected pores. Friction with the inner walls of the pores occurs, and ultimately the sound wave is dissipated as heat energy. As shown in Fig. 17, the study indicates that the porosity order of asphalt mixtures is as follows: OGFC-13 > SMA-13 > AC-13 = MS-Ш, and the noise reduction effect order is: OGFC-13 > SMA-13 > AC-13 > MS-Ш. This demonstrates that porous asphalt mixtures can effectively reduce noise [66]. Porous asphalt concrete also presents a better sound absorption coefficient than that of porous cement concrete with the same porosity in the high-frequency range of 1400–1550 Hz, due to its larger pores and more connected pores [67]. It is verified that the sound absorption coefficient of a porous mixture increases with the increase of porosity, whether in the low-frequency range or the high-frequency range [65,68]. Porous asphalt mixtures with higher radii, higher coordination numbers, characteristic pore sizes, and pore numbers lead to higher sound absorption coefficients. The sound absorption coefficient decreases as the void equivalent diameter increases due to viscosity loss and heat loss of sound energy. Thus, the sound absorption coefficient is closely related to porosity, characteristic pore size, and coordination number.

### 5.2 Impact of structure depth, material and thickness

**Fig. 18:**
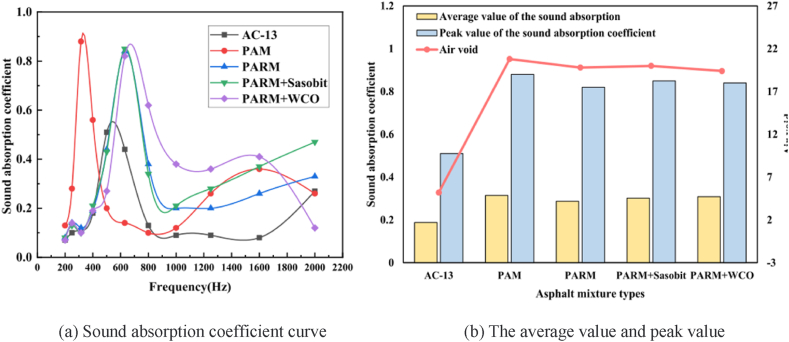
The sound absorption coefficient of asphalt mixture [65].

**Fig. 19:**
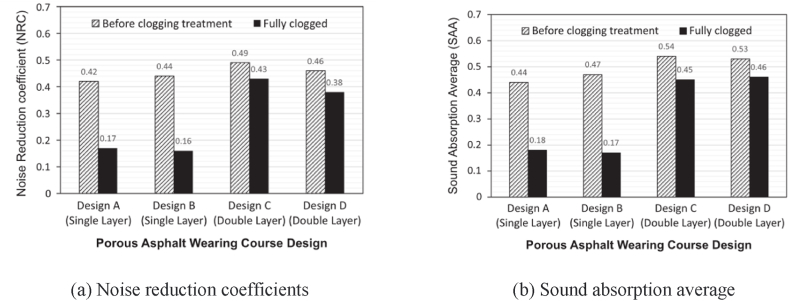
Comparison of sound absorption characteristics of porous asphalt designs [70].

Besides porosity, structural depth, materials, and structural thickness have different effects on the sound absorption coefficient of porous asphalt mixtures. The decrease in pavement noise can be explained by the phenomenon of Helmholtz resonance, which tends to occur in the cavities of the pavement surface with deep and rich texture depth and causes energy conversion from acoustic energy to heat energy and mechanical energy [67]. It has been verified that structural depth is inversely proportional to pavement noise [66,69]. Research also indicates that the noise reduction performance of porous pavement declines after long-term use, similar to dense-graded asphalt concrete [66]. The higher the structural depth, the better the sound absorption level of porous asphalt pavement. Meanwhile, structural depth also has a great correlation with the grading curve. This means that the sound level of the pavement surface improves with the increase of fine aggregates in the grading curve [69]. The damping properties of rubber are also beneficial to the sound absorption level of porous asphalt mixtures at frequencies of 500–1000 Hz, as shown in Fig. 18 [65]. The structural thickness of porous asphalt pavement also impacts its sound absorption performance. As shown in Fig. 19, the sound absorption capacity of double-layer porous pavement before plugging is slightly higher than that of single-layer porous pavement before clogging, but the sound absorption performance of double-layer pavement after clogging is obviously higher than that of single-layer pavement after clogging [70]. Some research also shows that the sound absorption level decreases with the increase of porous asphalt pavement thickness, as the peak value of the sound absorption coefficient of porous pavement moves to the low-frequency direction with the increase of thickness [68].

To optimize the sound absorption of porous asphalt mixtures, further exploration is needed on the ideal combination of structural depth and grading curve, and research on advanced anti-clogging materials and designs for double-layer pavements to maintain long-term sound absorption performance is essential.

## 6 Skid resistance

### 6.1 Impact of texture

**Fig. 20:**
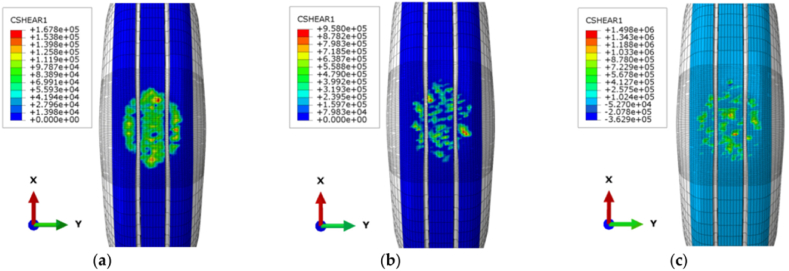
Cloud chart of shear force distribution on different pavements. (a) AC Pavement (b) SMA (c) OGFC Pavement [71].

As the tire-pavement contact decreases significantly under wet conditions, experimental studies and numerical models have been conducted by scholars to analyze the effect of water film thickness on the friction behaviors of porous asphalt pavement. The anti-skid performance of porous asphalt pavement is mainly attributed to its rough texture structure. Fig. 20 shows the influence of different pavement textures on tire-pavement friction behaviors based on the finite element analysis method in ABAQUS. The frictional force distribution between the tire and dense-graded asphalt mixture demonstrates a smooth surface, while the friction forces on stone mastic asphalt mixture and porous asphalt mixture, with relatively rough surfaces, are different. It is obvious that the protruding aggregate particles on the surface of the pavement are beneficial to the pavement texture properties. It is concluded that porous asphalt mixture containing more coarse aggregates than dense asphalt mixture and stone mastic asphalt mixture tends to have deeper texture depth and a rougher texture surface [71,72]. Moreover, porous asphalt mixtures with different nominal maximum sizes show different anti-skid performance of the pavement through different test methods. It was found that the skid resistance increased as the nominal maximum particle size increased through the sand patch volumetric test [73]. And other data also indicates that the friction coefficient of porous asphalt mixture with a larger nominal maximum particle size is lower than that of a smaller one by the British pendulum test [74]. The study also found that the Pendulum test value has a weak correlation with mean texture depth and the sand patch volumetric test reflects high-speed friction under wet conditions, while the British pendulum test reflects the general level of friction at low vehicle speeds [72,75]. Overall, sufficient texture depth contributes to the improvement of skid resistance to a certain extent [74,76,77].

The anti-skid performance of porous asphalt pavement is related to its rough texture and aggregate characteristics, and current test methods show inconsistent results regarding the impact of nominal maximum particle size, highlighting the need for a more comprehensive evaluation approach. Therefore, There is a need to develop a more comprehensive test method that combines the advantages of existing ones like the sand patch volumetric test and the British pendulum test, to accurately assess the anti-skid performance of porous asphalt pavement considering different aggregate sizes and texture depths. Also, research on modifying the surface texture of porous asphalt mixtures to enhance skid resistance without sacrificing other performance aspects should be intensified.

### 6.2 Impact of water film

A larger water film often results in worse skid resistance. The contact between the surface of the pavement and the tires will be incomplete if the pavement surface is covered with water and the tires are lifted by the water flow. The friction characteristics of the pavement are obviously reduced compared with those on dry conditions, which also greatly increases the incidence of traffic accidents. Thus, the water film on porous asphalt pavement is different under different weather conditions, which will impact the discharge of rainfall. Given a free rolling state and a braking state with a slip rate of 20 %, the vertical contact force between the tire and the pavement declines significantly with the increase in the thickness of the water film, and the maximum loss can account for 75 % of the applied vertical loads. Besides, as a superior pavement texture, it is demonstrated that the vertical contact force of porous asphalt pavement is the highest compared with SMA and AC under the condition of the same thickness of water film and a speed of 70 km/h [71]. Moreover, it is demonstrated that pavement with larger aggregates can decrease the water film thickness under the same, low rainfall intensity and porosity. The differences in water film thickness between different aggregate sizes are also found to be small at heavy rainfall intensities. This observation further indicates that porous asphalt pavements with larger-size aggregates for a given porosity exhibit better skid resistance performance [78].

The thickness of the water film affects porous asphalt pavement's skid resistance and vertical contact force with tires. Larger water films worsen skid resistance and reduce contact force. Porous asphalt pavement has better performance in some aspects like vertical contact force compared to others, and pavements with larger aggregates show better skid resistance under certain rainfall intensities.It's vital to further study how to optimize aggregate size and pavement texture to minimize the negative impact of water films and enhance skid resistance under various weather and rainfall conditions for safer traffic.

## 7 Conclusion

This paper comprehensively reviews the research progress on the durability, permeability, low-temperature performance, and functionality of porous asphalt mixtures. Various improvement measures and optimization suggestions are proposed, providing valuable references for future research and applications of porous asphalt mixtures. The conclusions drawn from this paper regarding porous asphalt mixture are as follows.

(1) SBS modified asphalt, rubber modified asphalt, or a combination of both are commonly used to enhance the interaction between the binder and aggregates, thereby improving the raveling resistance of porous asphalt mixtures.
(2) Fibers act as stabilizing additives that significantly increase the raveling resistance by increasing the asphalt film thickness and preventing asphalt leakage. Different types of fibers (e.g., polyester fibers, basalt fibers) exhibit varying effects, necessitating the selection of suitable fibers.
(3) The physical and chemical properties of aggregates, such as acid-base properties, water absorption coefficient, and Los Angeles Abrasion value, play crucial roles in the raveling resistance of porous asphalt mixtures. Alkaline aggregates, low water absorption, and low abrasion values are preferred to reduce susceptibility to raveling.
(4) Traffic loads and environmental conditions (e.g., temperature, moisture) significantly affect the raveling resistance of porous asphalt mixtures. Optimizing the mix design and incorporating stabilizing agents can effectively enhance durability.
(5) Rapid temperature variations lead to temperature gradients within porous asphalt mixtures, affecting their low-temperature performance. Frost heave damage caused by ice crystallization exacerbates structural damage.
(6) Higher porosity results in poorer low-temperature crack resistance. Thus, porous asphalt mixtures with smaller porosity are recommended for cold regions. Improvement Measures: Incorporating fibers (e.g., polyester fibers, basalt fibers) and steel slags can improve the low-temperature ductility and deformation resistance of porous asphalt mixtures.
(7) The permeability of porous asphalt mixtures is primarily influenced by pore structure, including porosity, pore connectivity, and pore size distribution. Optimizing pore structure to increase interconnected porosity is crucial for enhancing permeability.
(8) Long-term use leads to gradual clogging and reduced permeability. Improving mix design and drainage system design can delay clogging and maintain permeability.
(9) The large pore structure of porous asphalt mixtures effectively absorbs and disperses sound energy. Increasing porosity, texture depth, and using rubber asphalt can further enhance noise reduction.
(10) Skid resistance is influenced by texture depth, water film thickness, and additives. Larger texture depth and smaller water film thickness contribute to better skid resistance. Optimizing aggregate gradation and incorporating skid resistance additives can improve skid resistance.

Finally, some future research topics are recommended as follows.

(1) Seek an optimized solution for the balance among various performances to ensure improving one performance doesn't harm other essential performances.Further studies are needed to allow for the simultaneous improvement of multiple performance aspects, ensuring that the porous asphalt pavement can meet the diverse requirements of different traffic and environmental conditions with a well-balanced set of excellent properties.
(2) Deeply studies on various factors (like environment, traffic, materials, etc.) interacting and affecting the performance of porous asphalt mixtures should be developed. Building a more accurate prediction model is essential to support pavement design and material optimization.
(3) Further study on the feasibility of using industrial waste (like steel slag) as aggregates to cut costs and reduce pollution is recommended. Promote the application of porous asphalt pavement technology in environmental protection.

## CRediT authorship contribution statement

**Yue Zhou:** Writing – original draft, Validation, Software, Formal analysis, Conceptualization. **Zhiqiang Cheng:** Writing – review & editing, Visualization, Software, Conceptualization. **Xiaoguang Zheng:** Supervision, Resources, Methodology, Funding acquisition, Data curation. **Tao Wang:** Project administration, Funding acquisition, Formal analysis, Conceptualization. **Xiaoliang Wu:** Visualization, Validation, Resources, Data curation. **Xiaoxiao Yu:** Project administration, Methodology, Investigation, Conceptualization. **Shengjia Xie:** Software, Methodology, Funding acquisition, Conceptualization, Jie Lu, Writing – review & editing, Validation, Formal analysis.

## Declaration of competing interest

The authors declare that they have no known competing financial interests or personal relationships that could have appeared to influence the work reported in this paper.

## References

[1] Chu L., Fwa T.F. (2019). Functional sustainability of single- and double-layer porous asphalt pavements. Construct. Build. Mater..

[2] Lou Baowen, Sha Aimin, Barbieri Diego Maria, Liu Zhuangzhuang, Zhang Fan, Jiang Wei, Hoff Inge (2021). Characterization and microwave healing properties of different asphalt mixtures suffered freeze-thaw damage. J. Clean. Prod..

[3] Wu Jiantao, Wang Yihua, Liu Quan, Wang Yu, Ago Cadnel, Oeser Markus (2020). Investigation on mechanical performance of porous asphalt mixtures treated with laboratory aging and moisture actions. Construct. Build. Mater..

[4] Lastra-González Pedro, Indacoechea-Vega Irune, Calzada-Pérez Miguel A., Vega-Zamanillo Ángel, Castro-Fresno Daniel (2019). Assessment of induction heating in the performance of porous asphalt mixtures. Road Mater. Pavement Des..

[5] Clara Estéfani, Barra Breno Salgado, Teixeira Luiz Henrique, Mikowski Alexandre, Hughes Gary B., Nguyen Mai-Lan (2023). Influence of polymeric molecular chain structure on the rheological-mechanical behavior of asphalt binders and porous asphalt mixes. Construct. Build. Mater..

[6] Xu Lei, Ni Hangtian, Zhang Yi, Sun Daquan, Zheng Yunpeng, Hu Mingjun (2022). Porous asphalt mixture use asphalt rubber binders: preparation and noise reduction evaluation. J. Clean. Prod..

[7] Yan Kezhen, Sun Hao, You Lingyun, Wu Shenghua (2020). Characteristics of waste tire rubber (WTR) and amorphous poly alpha oleﬁn (APAO) compound modiﬁed porous asphalt mixtures. Construct. Build. Mater..

[8] Ren Shisong, Liu Xueyan, Xu Jian, Lin Peng (2021). Investigating the role of swelling-degradation degree of crumb rubber on CR/SBS modiﬁed porous asphalt binder and mixture. Construct. Build. Mater..

[9] Zhang Linyan, Ma Yong, Li Yanbin Zhao Bo, Li Peifeng, Xie Bin, Feng Jiliang (2021). Performance characteristics of epoxy modified open graded friction course (OGFC) by post-doping methods. J. Phys. Conf..

[10] Wang Sheng, Kang Aihong, Xiao Peng, Li Bo, Fu Weili (2019). Investigating the effects of chopped basalt fiber on the performance of porous asphalt mixture. Adv. Mater. Sci. Eng..

[11] Xiang Ma, Li Qiang, Cui Yu-Chao, Ni An-Qi (2018). Performance of porous asphalt mixture with various additives. Int. J. Pavement Eng..

[12] Zhanga Kun, Lim Justin, Nassiri Somayeh, Englundb Karl, Li Hui (2019). Reuse of carbon ﬁber composite materials in porous hotmix asphalt to enhance strength and durability. Case Stud. Constr. Mater..

[13] Li Mingliang, Ren Shisong, Liu Xueyan, Wu Zhe, Zhang Haopeng, Fan Weiyu, Lin Peng, Xu Jian (2022). A comprehensive study on the rejuvenation efficiency of compound rejuvenators for the characterization of the bituminous binder, mortar, and mixture. Materials.

[14] Xu Bin, Ding Runduo, Yang Zhihao, Sun Yiren, Zhang Jiancheng, Lu Kaiji, Cao Dongwei, Gao Aodong (2023). Investigation on performance of mineral-oil-based rejuvenating agent for aged high viscosity modified asphalt of porous asphalt pavement. J. Clean. Prod..

[15] Ren Jiaolong, Xu Yinshan, Huang Jiandong, Wang Yi, Ji Zhirong (2021). Gradation optimization and strength mechanism of aggregate structure considering macroscopic and mesoscopic aggregate mechanical behavior in porous asphalt mixture. Construct. Build. Mater..

[16] Xu Guangji, Fan Jianwei, Ma Tao, Zha Wei, Ding Ximao, Wang Zhiwen (2021). Research on application feasibility of limestone in sublayer of Double-Layer permeable asphalt pavement. Construct. Build. Mater..

[17] Song Weimin, Xu Fei, Wu Hao, Xu Zihao (2021). Investigating the skeleton behaviors of open-graded friction course using discrete element method. Powder Technol..

[18] Jin Dongzhao, Meyer Theresa K., Chen Siyu, Ampadu Boateng Kwadwo, Pearce Joshua M. (2022). Zhanping You. Evaluation of lab performance of stamp sand and acrylonitrile styrene acrylate waste composites without asphalt as road surface materials. Construct. Build. Mater..

[19] Hong Zhan, Kumar Anupam, Athanasios Skarpas, Cor Kasbergen, and Sandra Erkens. Simple homogenization-based approach to predict raveling in porous asphalt, Transport. Res. Rec. 2674(12).

[20] Lucas Yu Pingzhou, James Tsai Yichang (2022). Quantifying Raveling Using 3D technology with loss of aggregates as a new performance indicator. Sustainability.

[21] Sangiorgi Cesare, Eskandarsefat Shahin, Tataranni Piergiorgio, Simone Andrea, Vignali Valeria, Lantieri Claudio, Dondi Giulio (2017). Acomplete laboratory assessment of crumb rubber porous asphalt. Construct. Build. Mater..

[22] Shukrya Nurul Athma Mohd, Abdul Hassana Norhidayah, Hainina Mohd Rosli, Abdullahb Mohd Ezree, Abdullaha Nor Asniza Mohamed, Mahmuda Mohd Zul Hanif, Putrajayaa Ramadhansyah, Mashros Nordiana (2016). Experimental evaluation of antistripping additives on porous asphalt mixtures. Jurnal Teknologi (Sciences & Engineering).

[23] Cheng Zhihao, Zheng Shaopeng, Liang Naixing, Li Xiao, Li Libin (2023). Inﬂuence of complex service factors on ravelling resistance performance for porous asphalt pavements. Buildings.

[24] Laura Manrique S. (2015).

[25] Namaa Mohammed M., Qasim Zaynab I., Ibrahim AlHelo Karim H. (2021). Study of the properties of open graded asphalt mixtures with the addition of SBS. Mater. Sci. Eng..

[26] Zhang Jiawei, Huang Weidong, Zhang Yuan, Qi Cai, Yan Chuanqi, Lv Quan (2021). Investigation on the durability of OGFC-5 ultra-thin friction course with different mixes. Construct. Build. Mater..

[27] Cetin Altan (2013). Effects of crumb rubber size and concentration on performance of porous asphalt mixtures. International Journal of Polymer Science.

[28] Jiao Yubo, Zhang Yao, Fu Liuxu, Guo Meng, Zhang Lidong (2019). Influence of crumb rubber and tafpack super on performances of SBS modified porous asphalt mixtures. Road Mater. Pavement Des..

[29] Sangiorgi Cesare, Shahin Eskandarsefat ⇑, Tataranni Piergiorgio, Simone Andrea, Vignali Valeria, Lantieri Claudio, Dondi Giulio (2017). Acomplete laboratory assessment of crumb rubber porous asphalt. Construct. Build. Mater..

[30] Cheng Yongchun, Chai Chao, Zhang Yuwei, Chen Yu, Zhu Bing (2019). A new eco-friendly porous asphalt mixture modiﬁed by crumb rubber and basalt fiber. Sustainability.

[31] Zhang Jiawei, Huang Weidong, Zhang Yuan, Lv Quan, Yan Chuanqi (2020). Evaluating four typical ﬁbers used for OGFC mixture modiﬁcation regarding drainage, raveling, rutting and fatigue resistance. Construct. Build. Mater..

[32] Chen Jian-Shih, Chen Shih-Fan, Liao Min-Chih (2014). Laboratory and field evaluation of porous asphalt concrete. Asian Transport Studies.

[33] Gupta Anik, Lastra-Gonzalez Pedro, Castro-Fresno Daniel, Rodriguez-Hernandez Jorge (2021). Laboratory characterization of porous asphalt mixtures with aramid fibers. Materials.

[34] Gupta Anik, Lastra-Gonzalez Pedro, Rodriguez-Hernandez Jorge, González María González, Castro-Fresno Daniel (2021). Critical assessment of new polymer-modified bitumen for porous asphalt mixtures. Construct. Build. Mater..

[35] Çetin Altan, Oral Gökhan (2022). Performance evaluation of porous asphalt mixtures modified with basalt fiber. Revista de la Construcción..

[36] Lin Peng, Liu Xueyan, Ren Shisong, Yi Li, Xu Jian, Li Mingliang (2023). Unraveling the influence of fibers on aging susceptibility and performance of high content polymer modified asphalt mixtures. Case Stud. Constr. Mater..

[37] Zhanga Kun, Limb Justin, Nassirib Somayeh, Englundb Karl, Li Hui (2019). Reuse of carbon fiber composite materials in porous hot mix asphalt to enhance strength and durability. Case Stud. Constr. Mater..

[38] Lin Qi, Yu Baoyang, Song Jingang (2022). Freeze-Thaw damage characteristics of composite modified open graded friction course. Frontiers in Materials.

[39] Shukrya Nurul Athma Mohd, Abdul Hassana Norhidayah, Hainina MohdRosli, Abdullahb Mohd Ezree, Abdullaha Nor Asniza Mohamed, Mahmuda Mohd Zul Hanif, Putrajayaa Ramadhansyah, Mashros Nordiana (2016). Experimental evaluation of antistripping additives on porous asphalt mixtures. Jurnal Teknologi (Sciences & Engineering).

[40] Patricia Pérez Fortes Ana, Anastasio Sara, Kuznetsova Elena, Danielsen Svein Willy (2016). Behaviour of crushed rock aggregates used in asphalt surface layer exposed to cold climate conditions. Environ. Earth Sci..

[41] Herndon David A., Xia Feipeng, Amirkhanian Serji, Wang Hao (2016). Investigation of Los Angeles value and alternate aggregate gradations in OGFC mixtures. Construct. Build. Mater..

[42] Haripriya Nekkanti, Bradley J. Putman, Behrooz Danish. Influence of aggregate gradation and nominal maximum aggregate size on the performance properties of OGFC mixtures, Transport. Res. Rec. 2673(1).

[43] Zhu Bing, Liu Hanbing, Li Wenjun (2020). Fracture behavior of permeable asphalt mixtures with steel slag under low temperature based on acoustic emission technique. Sensors.

[44] Pathak Santanu, Asce S.M., Choudhary Rajan, Asce AffM., Kumar Abhinay, Asce S.M., Kumar Shukla Sanjay, Asce M. (2020). Evaluation of benefits of open-graded friction courses with basic oxygen furnace steel-slag aggregates for hilly and high-rainfall regions in India. J. Mater. Civ. Eng..

[45] Zhu Bing, Liu Hanbing, Li Wenjun (2020). Fracture behavior of permeable asphalt mixtures with steel slag under low temperature based on acoustic emission technique. Sensors.

[46] Chai Chao, Cheng Yongchun, Zhang Yuwei, Zhu Bing, Liu Hang (2020). Mechanical properties of crumb rubber and basalt fiber composite modified porous asphalt concrete with steel slag as aggregate. Polymers.

[47] Zheng Chuanfeng, Xu Junpeng, Zhang Ting, Tan Guojin (2021). Study on the microscopic damage of porous asphalt mixture under the combined action of hydrodynamic pressure and ice crystal frost heave. Construct. Build. Mater..

[48] Liu Denggao, Zhang Haitao, Yu Tengjiang (2022). etal. Meso-structural characteristics of porous asphalt mixture based on temperature-stress coupling and its influence on aggregate damage. Construct. Build. Mater..

[49] Wu Jinrong, Li Fei, Ma Qinyong (2020). Effect of polyester fiber on air voids and low-temperature crack resistance of permeable asphalt mixture. Adv. Civ. Eng..

[50] Zhang Zhengwei, Sha Aimin, Liu Xiang, Luan Bo, Gao Jie, Jiang Wei, Ma Feng (2020). State-of-the-art of porous asphalt pavement: experience and considerations of mixture design. Construct. Build. Mater..

[51] Guo Fucheng, Li Rui, Lu Shuhua, Bi Yanqiu, He Haiqi (2020). Evaluation of the eﬀect of fiber type, length, and content on asphalt properties and asphalt mixture performance. Materials.

[52] Zhang Kun, Liu Yilong, Nassiri Somayeh (2021). Performance evaluation of porous asphalt mixture enhanced with high dosages of cured carbon fiber composite materials. Construct. Build. Mater..

[53] Lou Baowen, Liu Zhuangzhuang, Sha Aimin, Jia Meng, Li Yupeng (2020). Microwave absorption ability of steel slag and road performance of asphalt mixtures incorporating steel slag. Materials.

[54] Zhang Tao, Wu Jinrong, Hong Rongbao, Ye Shupeng, Jin Aihua (2022). Research on low-temperature performance of steel slag/polyester fiber permeable asphalt mixture. Construct. Build. Mater..

[55] Hu Jianying, Tao Ma a, Kang Ma (2021). DEM-CFD simulation on clogging and degradation of air voids in double-layer porous asphalt pavement under rainfall. J. Hydrol..

[56] Jin Dongzhao, Ge Dongdong, Chen Siyu, Che Tiankai, Liu Hongfu, Malburg Lance, You Zhanping (2021). Cold in-place recycling asphalt mixtures: laboratory performance and preliminary M-E design analysis. Materials.

[57] Jin Dongzhao, Yin Lei, Malburg Lance, You Zhanping (2024). Laboratory evaluation and field demonstration of cold in-place recycling asphalt mixture in Michigan low-volume road. Case Stud. Constr. Mater..

[58] Meng Anxin, Xing Chao, Tan Yiqiu, Xiao Shenqing, Li Jilu, Li Guannan (2020). Investigation on clogging characteristics of permeable asphalt mixtures. Construct. Build. Mater..

[59] Zhan Haitao, Liu Zuoqiang, Yu Lize, Sun Quansheng (2020). Study on microstructure influence mechanism to mechanical behavior of OGFC asphalt mixture. Can. J. Civ. Eng..

[60] Hu Mingjun, Li Lihan, Peng Fangxing (2019). Laboratory investigation of OGFC-5 porous asphalt ultra-thin wearing. Construct. Build. Mater..

[61] Chen Jun, Wang Junpeng, Wang Hao, Asce M., Xie Pengyu, Guo Lukai (2020). Analysis of pore characteristics and flow pattern of open-graded asphalt mixture in different directions. J. Mater. Civ. Eng..

[62] Meng Anxin, Xing Chao, Tan Yiqiu, Xiao Shenqing, Li Jilu, Li Guannan (2020). Investigation on clogging characteristics of permeable asphalt mixtures. Construct. Build. Mater..

[63] Ji Tianjian, Xiao Lei, Chen Feng (2020). Parametric analysis of the drainage performance of porous asphalt pavement based on a 3D FEM method. J. Mater. Civ. Eng..

[64] Bamunuarachchi B.P.D.P.P., Mampearachchi W.K. (2020). Investigation of durability of open graded friction courses. Transport. Res. Procedia.

[65] Xu Lei, Ni Hangtian, Zhang Yi, Sun Daquan, Zheng Yunpeng, Hu Mingjun (2022). Porous asphalt mixture use asphalt rubber binders: preparation and noise reduction evaluation. J. Clean. Prod..

[66] Lai Feng, Huang Zhiyong, Guo Feng (2021). Noise reduction rharacteristics of macroporous asphalt pavement based on a weighted sound pressure level sensor. Materials.

[67] Song Weimin, Zhang Miaomiao, Wu Hao, Liu Zhuo, Yin Jian (2023). Effect of pore characteristics on sound absorption of permeable pavement materials. Adv. Civ. Eng..

[68] Wang Zhanqi, Xie Jianguang, Gao Lei, Liu Mingxi, Liu Yanping (2020). Improvement of acoustic model and structural optimization design of porous asphalt concrete based on meso-structure research. Construct. Build. Mater..

[69] Abbassi Saeed, Kazemi Muhammad, Norouzi Nima, Nasiri Zahra (2021). Impacts of vehicle tire on slip resistance and sound pollution in asphalt pavements. Brilliant Engineering.

[70] Chu L., Fwa T.F. (2019). Functional sustainability of single- and double-layer porous asphalt pavements. Construct. Build. Mater..

[71] Miao Yu, Kong Yao, You Zhanping, Li Jue, Yang Liming (2022). Anti-skid characteristics of asphalt pavement based on partial tire aquaplane conditions. Materials.

[72] Yu Bin, Jiao Liya, Ni Fujian, Yang Jun (2015). Long-term ﬁeld performance of porous asphalt pavement in China. Road Mater. Pavement Des..

[73] Zhang Haitao, Liu Zuoqiang, Yu Lize, Sun Quansheng (2021). Study on microstructure influence mechanism to mechanical behavior of OGFC asphalt mixture. Can. J. Civ. Eng..

[74] Hu Yuanjiao, Sun Zhaoyun, Han Yuxi, Li Wei, Pei Lili (2022). Evaluate pavement skid resistance performance based on Bayesian-LightGBM using 3D surface macrotexture data. Materials.

[75] Marcia Lopes Afonso, Dinis-Almeida Marisa, Fael Cristina Sena (2019). Characterization of the skid resistance and mean texture depth in a permeable asphalt pavement. Mater. Sci. Eng..

[76] Marcia Lopes Afonso, Dinis-Almeida Marisa, Fael Cristina Sena (2019). Characterization of the skid resistance and mean texture depth in a permeable asphalt pavement. Mater. Sci. Eng..

[77] Zhu Xingyi, Yang Yang, Zhao Hongduo, Jelagin Denis, Chen Feng, Gilabert Francisco A., Guarin Alvaro (2021). Effects of surface texture deterioration and wet surface conditions on asphalt runway skid resistance. Tribol. Int..

[78] Zhang Lei, Ong Ghim Ping, Tien Fang F.W.A. (2014). Numerical study on the influence of aggregate size on skid resistance performance of porous pavements. Asian Transport Studies.

